# Effect of Liquid Smoke Incorporation on the Structural, Barrier, and Functional Properties of Okra Mucilage–Corn Starch Films

**DOI:** 10.3390/polym18131566

**Published:** 2026-06-23

**Authors:** Nayanne Lima Dos Santos Ferreira, Luana Kelly Sampaio Facundo, Maryana Melo Frota, Maria Do Socorro Rocha Bastos, Lorena Maria Freire, Kaliana Sitônio Eça, Jeanlex Soares de Sousa, João Borges Laurindo, Thomas Karbowiak, Patrícia Marques De Farias, Markus Schmid, Luciana De Siqueira Oliveira

**Affiliations:** 1 Department of Food Engineering, Center of Agrarian Sciences, Federal University of Ceará, Pici Campus, Fortaleza 60455-760, Ceará, Brazil; nayannelims@hotmail.com (N.L.D.S.F.); luanafacundo@alu.ufc.br (L.K.S.F.); mary.m.frota@gmail.com (M.M.F.); freire278@gmail.com (L.M.F.); kaliana.se@ufc.br (K.S.E.); 2 Embrapa Agroindústria Tropical, Rua Dra. Sara Mesquita, 2270, Planalto Pici, Fortaleza 60511-110, Ceará, Brazil; socorro.bastos@embrapa.br; 3 Department of Physics, Center of Sciences, Federal University of Ceará, Pici Campus, Fortaleza 60455-970, Ceará, Brazil; jeanlex@fisica.ufc.br; 4 Department of Chemical and Food Engineering, Technological Center, Federal University of Santa Catarina, Floria-nópolis 88040-900, Santa Catarina, Brazil; joao@enq.ufsc.br; 5 Procédés Alimentaires et Microbiologiques (UMR PAM), Institut Agro, Université Bourgogne Europe (INRAE), 21000 Dijon, France; thomas.karbowiak@agrosupdijon.fr; 6 Sustainable Packaging Institute SPI, Faculty of Life Sciences, Albstadt-Sigmaringen University, Anton-Günther-Straße 51, 72488 Sigmaringen, Germany; marquesdefarias@hs-albsig.de

**Keywords:** okra mucilage, liquid smoke, biopolymer film, antioxidant activity, barrier properties

## Abstract

The present study investigated the effect of liquid smoke (LS) on the physicochemical, structural, barrier, and functional properties of okra mucilage–corn starch (OMCS) films. Formulations containing varying concentrations of LS (0–3%) were prepared using the casting method. The incorporation of LS modified the rheological behavior of the film-forming dispersions, as evidenced by increased apparent viscosity and consistency index. In the films, water solubility increased from 43.6 to 53.2%, contact angle increased from 31.9° to 55.6°, and opacity increased from 4.73 to 8.83, while water vapor permeability decreased from 1.05 to 0.88 g·mm·m^−2^·h^−1^·kPa^−1^, indicating modifications in matrix organization and surface hydrophobicity. Tensile strength increased from 26.3 to 40.5 MPa at 3% LS, accompanied by a slight reduction in elongation, suggesting enhanced structural rigidity. Structural analyses revealed interactions between the LS phenolic compounds and the polysaccharide hydroxyl groups, resulting in a more cohesive polymeric network. LS was the main contributor to the film’s antioxidant activity owing to its elevated phenolic content and free radical scavenging capacity. The films also showed substantial degradation under soil burial conditions, with mass loss ranging from 61% to 96%. Overall, LS proved to be an effective functional additive, improving the structural and antioxidant performance of OMCS films and expanding their potential for active food packaging applications.

## 1. Introduction

The use of biopolymer films as an alternative to conventional plastic packaging has attracted increasing attention from the food industry and researchers, mainly due to their potential to minimize environmental impacts and preserve food quality [1,2]. These films, made from natural biopolymers such as proteins, starch, and polysaccharides, exhibit desirable properties such as biocompatibility, flexibility, and transparency [3,4].

Among plant-derived biopolymers, okra mucilage (*Abelmoschus esculentus* L.) stands out for its unique properties. This hydrophilic polysaccharide is primarily composed of D-galactose, L-rhamnose, and galacturonic acid, thereby forming cohesive, stable structures [5,6]. Additionally, its wide availability and low cost enhance its technological viability [7]. As demonstrated in research conducted by Araújo et al. [8], films developed with okra mucilage and corn starch exhibited a homogeneous surface, adequate mechanical resistance, and low water vapor permeability. These observations suggest that this biopolymer has potential for use in packaging materials.

Liquid smoke (LS) is obtained by condensing the vapors generated in the controlled pyrolysis of lignocellulosic biomass, such as wood, coconut husk, and other plant residues. It is widely used in the food industry as a natural additive due to its antioxidant and flavoring properties [1,9]. Its composition, rich in phenolic compounds, carbonyls, and organic acids, enables chemical interactions with polymeric matrices, thereby improving the mechanical and barrier properties of edible films and coatings [10]. The application of LS in food has demonstrated potential for preserving fish and seafood [2], tofu [9] and processed meat [1], thereby reducing lipid oxidation and inhibiting microbial growth. Furthermore, its incorporation into edible coatings based on starch and chitosan has been associated with improvements in mechanical and barrier properties, reinforcing its potential for the development of active and biodegradable packaging [1,10,11].

Despite the growing interest in starch–mucilage biopolymer films, these systems still present limitations related to their hydrophilic nature and moisture sensitivity, which may compromise water resistance and barrier performance [8]. In this context, LS has emerged as a promising additive due to its antioxidant properties and its potential to interact with hydroxyl-rich polysaccharide matrices through phenolic and carbonyl compounds [1,10,11].

Although LS has been incorporated into starch- and chitosan-based films and coatings [1,9,10,11], its effects on okra mucilage–corn starch films remain unclear, particularly regarding the relationship between matrix organization, hydrophilicity, and barrier properties. Therefore, the hypothesis of this study is that incorporating LS into okra mucilage–corn starch (OMCS) films can simultaneously enhance antioxidant activity and modify the structural organization of the polymeric matrix, thereby altering rheological, mechanical, barrier, and surface properties. Based on this hypothesis, the present study evaluated the effect of LS incorporation on the physicochemical, structural, barrier, antioxidant, and biodegradation properties of OMCS films.

## 2. Materials and Methods

The LS used in this study was commercially sourced from DaRoca^®^ (Caucaia, CE, Brazil), Brazil. The corn starch was purchased from a local market in Fortaleza, Ceará, Brazil. Glycerol was purchased from Merck.

Okra (*Abelmoschus esculentus* L.) was purchased from a local market in Fortaleza, Ceará, Brazil. The plant material was used in accordance with national legislation and the Convention on Biological Diversity. No specific permits were required for the use of okra fruit in this study.

### 2.1. Extraction of Okra Mucilage

Okra mucilage was extracted and purified as described by Ghori et al. [12]. The okra (1.2 kg), at the mature stage, was washed with running water to remove dirt, sanitized in a chlorinated solution (100 ppm), and cut into 1 cm thick slices. It was then mixed with distilled water (1:3 ratio) in a plastic container and refrigerated at 10 ± 2 °C for 12 h. The mixture was then filtered through a 200-mesh nylon cloth, after which the mucilage was precipitated with a 1:2 solution of analytical-grade ethanol and water. The precipitated material was macerated for 30 min at 25 °C, then purified by solubilization, filtration through nylon (200 mesh), and washing with analytical-grade acetone. After drying for 24 h at 40 °C, the product (520.0 mg/kg, 0.06% efficiency on a fresh weight basis) was packaged in a plastic container and stored in a desiccator at room temperature (25 ± 2 °C, 50% relative humidity).

The material was characterized using Fourier-Transform Infrared Spectroscopy (FTIR).

### 2.2. Film Development

The films were prepared following the casting method described by Araújo et al. [8] with minor modifications. Briefly, a film-forming dispersion (FFD) was prepared by homogenizing a colloidal system, casting it onto a 30 × 44 cm glass plate with a 1.0 mm aluminum casting rod, and drying it at room temperature.

The base FFD consisted of 3 g of corn starch, 0.53 g of glycerol, 10 mL of distilled water, and 40 mL of okra mucilage. These proportions were maintained constant for all samples. Initially, starch, glycerol, and distilled water were homogenized at 100 rpm for 2 h at 25 °C. Subsequently, okra mucilage was incorporated, and the mixture was heated to 60 °C and maintained at this temperature for 25 min. LS was then added at concentrations of 0, 1, 2, and 3% (*v*/*v*) relative to the volume of okra mucilage, yielding four formulations designated F0, F1, F2, and F3, respectively. The concentration of LS was the sole experimental variable. After complete dispersion, the FFDs were cast onto glass plates and dried at room temperature (25 ± 2 °C) for 24 h.

**Figure 1:**
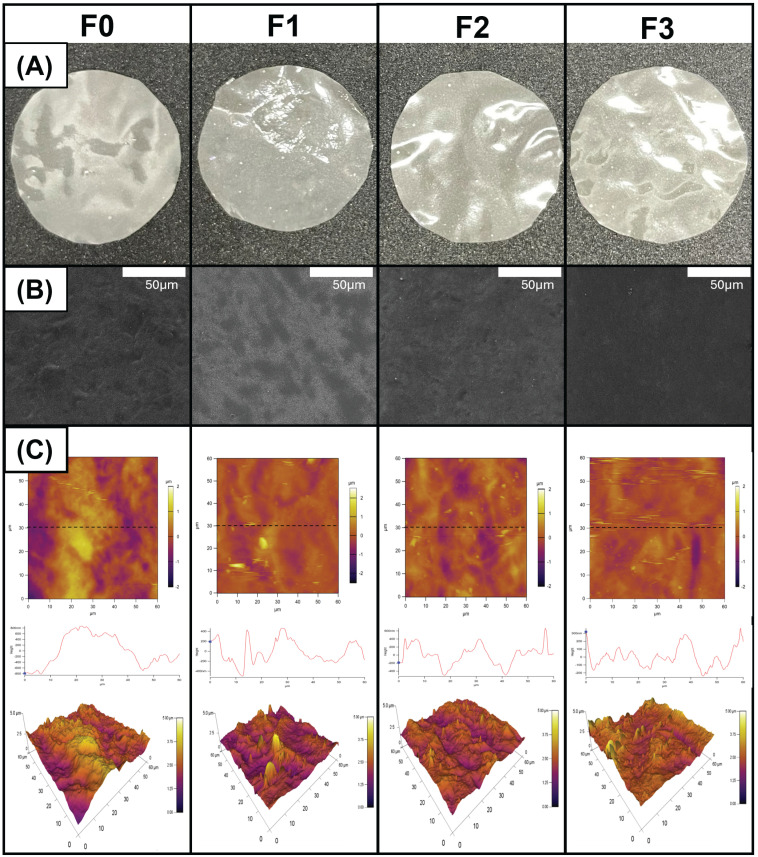
Visual appearance and surface morphology of OMCS films containing different concentrations of liquid smoke (LS). (**A**) Photographs of the films; (**B**) scanning electron microscopy (SEM) micrographs; and (**C**) atomic force microscopy (AFM) images, including topographic maps, surface profiles, and three-dimensional reconstructions. F0, F1, F2, and F3 correspond to formulations containing 0%, 1%, 2%, and 3% LS, respectively.

The resulting OMCS films were carefully peeled from the plates and subsequently characterized. Representative OMCS films are shown in Figure 1.

### 2.3. Rheological Characterization of FFDs

The rheological behavior of the FFD was evaluated using a concentric cylinder rheometer (Brookfield Searle, R/S SST Plus 2000; Brookfield Engineering Laboratories, Middleboro, MA, USA) at 22–25 °C. Apparent viscosity, shear stress, and shear rate were recorded over a shear rate range of 0–200 s^−1^ (ascending curve), followed by 200–0 s^−1^ (descending curve). Each measurement was conducted over 1 min, with 25 data points collected per curve.

All measurements were performed in triplicate using independent samples for each run. The pH of the film-forming dispersions was measured at 22–25 °C using a digital pH meter (Q400A, Quimis, Diadema, SP, Brazil).

The flow behavior was modeled using the Power Law (Ostwald–de Waele) model, which provided the best fit to the experimental data, as expressed in Equation (1):

(1) $$Power\;Law:\tau =K({\gamma )}^{n}$$

where τ is the shear stress (Pa), K is the consistency index coefficient of the Power Law (Pa·s^n^), γ is the shear rate (s^−1^), and n is the flow behavior index (dimensionless).

### 2.4. OMCS Film Characterization

#### 2.4.1. Film Thickness

Film thickness was measured at eight random positions along each sample using a digital micrometer (Mitutoyo^®^; Mitutoyo Corporation, Kawasaki, Kanagawa, Japan) with a precision of 0.001 mm. Measurements were conducted under controlled environmental conditions (25 ± 3 °C and 50 ± 6% relative humidity).

#### 2.4.2. Moisture Content

Moisture content was determined according to AOAC methods [13]. Approximately 1.0 g of each film sample was weighed into pre-dried crucibles and dried in a forced-air circulation oven (Solab, SL-102; Solab Científica, Piracicaba, SP, Brazil) at 105 °C for 6 h until constant mass was achieved. Analyses were performed in quintuplicate for each formulation.

#### 2.4.3. Water Solubility

Film solubility in water was determined according to the method described by Wang et al. [14] with minor modifications. Circular film samples (30 mm in diameter) were initially dried at 105 °C for 24 h in a forced-air circulation oven (Solab, SL-102; Solab Científica, Piracicaba, SP, Brazil) to obtain the initial dry mass. Each dried sample was then immersed in 50 mL of distilled water in an Erlenmeyer flask, sealed with Parafilm^®^ M (Parafilm®; Amcor/Bemis, Neenah, WI, USA), and incubated at 25 °C under agitation (75 rpm) in a shaker incubator (Solab, model SL 222) for 24 h. After immersion, the samples were recovered by filtration, dried again at 105 °C to constant mass, and weighed to determine the final dry mass (Wf).

All measurements were performed in quintuplicate for each formulation. Water solubility (%) was calculated using Equation (2):

(2) $$Solubility\left(\%\right)=[\left(W0-Wf\right)/W0]\times 100$$

where W0 is the initial dry mass of the sample before immersion, and Wf is the final dry mass after immersion.

#### 2.4.4. Water Vapor Permeability (WVP)

WVP was determined according to the standard method described in ASTM E96 [15]. Film samples were sealed over acrylic permeation cells containing 2 mL of distilled water, providing an exposed area of approximately 15.2 cm^2^. The cells were placed in a controlled-environment desiccator (ARSEC, DC-040; Arsec Dehumidifiers, SP, Brazil) at 25 ± 5 °C and 70 ± 5% relative humidity.

The mass gain of each cell was recorded over time, and measurements were performed in quintuplicate for each formulation. WVP (g·mm·m^−2^·h^−1^·kPa^−1^) was calculated according to Equation (3):

(3) WVP=(Δm/Δt)·(T/(A·ΔP))

where Δm/Δt is the steady-state water vapor transmission rate (g·h^−1^), T is the film thickness (mm), A is the exposed film area (m^2^), and ΔP is the partial water vapor pressure difference across the film (kPa).

#### 2.4.5. Mechanical Properties

The tensile strength (TS) and elongation at break (EB) of the OMCS films were determined according to ASTM D882-09 [16] using a universal testing machine (EMIC DL-3000; Instron/EMIC, PR, Brazil). Rectangular specimens (60 × 12 mm) were prepared and conditioned at 50 ± 5% relative humidity and 24 ± 2 °C for 48 h in a desiccator containing magnesium nitrate hexahydrate.

Prior to testing, specimens were mounted between grips with an initial gauge length of 60 mm. Tests were performed using a 100 N load cell at a crosshead speed of 50 mm·min^−1^. Force (N) and elongation (mm) were continuously recorded until sample failure. Seven independent replicates were analyzed for each formulation.

TS (MPa) was calculated as the maximum force at break divided by the initial cross-sectional area of the specimen. EB (%) was determined as the percentage increase in length at the point of rupture relative to the initial gauge length.

#### 2.4.6. Contact Angle (CA)

CA was determined in accordance with ASTM D5725-99 [17] using a DataPhysics OCA contact angle system (DataPhysics Instruments GmbH, Filderstadt, Germany). Measurements were performed at room temperature (25 °C) by depositing approximately 6 μL of distilled water onto the surfaces of film samples (10 × 10 mm) fixed to glass slides with double-sided adhesive tape. For each film formulation, ten independent measurements were conducted at different locations on the sample surface, and the reported values correspond to the average contact angle.

#### 2.4.7. Optical Properties

Color parameters were determined using the CIE L*a*b* color space, in which L* represents lightness (0 = black; 100 = white), a* denotes the green (−a*) to red (+a*) axis, and b* corresponds to the blue (−b*) to yellow (+b*) axis. Measurements were performed using a colorimeter (Konica Minolta CR-400, Konica Minolta Inc., Tokyo, Japan).

The total color difference (ΔE*) between the samples and the reference (white standard and/or control sample) was calculated according to Equation (4):

(4) $$\Delta E^{*}=\surd [{\left(L_{0}*-L*\right)}^{2}+{\left(a_{0}*-a*\right)}^{2}+{\left(b_{0}*-b*\right)}^{2}]$$

where L_0_*, a_0_*, and b_0_* correspond to the color parameters of the reference standard [18].

The whiteness index (WI) and yellowness index (YI) were calculated according to ASTM E3013-00 [19] using Equations (5) and (6), respectively:

*WI* = 100 − √[(100 − L*)^2^ + a*^2^ + b*^2^] (5)

(6) $${YI=YI=142.86\cdot b*/L*}^{*2}\;$$

The optical properties of the OMCS films were evaluated using a UV–visible spectrophotometer (UV-160A, Shimadzu Corporation, Kyoto, Japan). Film samples were cut into rectangular strips (1 × 5 cm) and placed in spectrophotometric cuvettes, with the film surface oriented perpendicular to the incident light beam.

The absorbance at 550 nm (A_550_) was recorded for each sample and used to determine film opacity, following the method proposed by Shojaee-Aliabadi et al. [20], as described in Equation (7).

(7) $$Opacity:\;OP=A_{550}/FT$$

where A_550_ is the absorbance at 550 nm, and FT is the film thickness (mm).

Film transparency was evaluated by transmittance at 600 nm (T_600_), as described by Han et al. [21], as expressed in Equation (8):

(8) $$Transparency:TP=T_{600}/FT$$

where T_600_ is the transmittance at 600 nm (%), and FT is the film thickness (mm).

#### 2.4.8. Fourier-Transform Infrared Spectroscopy (FTIR) Analysis of OMCS Films and Precipitated Material

FTIR spectra of the film samples were recorded using an IRTracer-100 spectrophotometer (Shimadzu Corporation, Kyoto, Japan). Measurements were performed in attenuated total reflectance (ATR) mode over the spectral range of 4000–400 cm^−1^, with 64 scans and a resolution of 4 cm^−1^.

The precipitated material was analyzed using the same instrument, employing the potassium bromide (KBr) pellet method under identical spectral conditions.

#### 2.4.9. Surface Morphology

The surface morphology of the OMCS films was examined by scanning electron microscopy (SEM) and atomic force microscopy (AFM). SEM analysis was performed using a Zeiss DSM 940A microscope (Carl Zeiss, Oberkochen, Germany). Prior to analysis, the samples were sputter-coated with a thin layer of gold (Electron Microscopy Sciences, Hatfield, PA, USA) to enhance conductivity. The micrographs were obtained at an accelerating voltage of 20 kV and a magnification of 2000×.

AFM analyses were performed using an MFP-3D-BIO atomic force microscope (Asylum Research, Santa Barbara, CA, USA) coupled to an inverted optical microscope (Nikon IX51, Nikon Corporation, Tokyo, Japan). Silicon nitride cantilevers with pyramidal tips (TR800PSA, Olympus, Tokyo, Japan) and a nominal spring constant of 0.15 N m^−1^ were used. Images were acquired in contact mode at a scan rate of 0.1 Hz and room temperature (25 °C).

#### 2.4.10. Antioxidant Activity and Total Extractable Polyphenols (TEP)

For the determination of total extractable polyphenols (TEP) and antioxidant activity (ABTS and DPPH assays), extracts were prepared from LS and OMCS film samples. Briefly, 100 mg of film or 100 μL of LS was added to 10 mL of 50% (*v*/*v*) ethanol, and the mixture was stirred magnetically for 1 h. The mixtures were subsequently sonicated in an ultrasonic bath (Cristófoli, 170 W, 42 kHz, Brazil) for 30 min and centrifuged at 17,709× *g* for 20 min at 4 °C. The resulting supernatants were collected and used for further analysis.

##### TEP

The TEP content of the extracts was determined according to the method of Obanda et al. [22] with slight modifications. An aliquot of 250 μL of the extract was mixed with 250 μL of Folin–Ciocalteu reagent (10%, *v*/*v*). Then, 500 μL of sodium carbonate solution (20%, *w*/*v*) and 500 μL of distilled water were added. The reaction mixtures were incubated in the dark at room temperature for 30 min, and absorbance was measured at 700 nm using a microplate reader (SynergyMx, BioTek Instruments, Winooski, VT, USA).

Quantification was performed using a gallic acid standard curve (0–50 μg mL^−1^; y = 0.020x + 0.009; R^2^ = 0.997), and the results were expressed as mg gallic acid equivalents (GAE) per g of LS or film.

##### Antioxidant Activity

For the DPPH assay, 500 μL of the extract was mixed with 500 μL of a DPPH radical solution (0.1 mM). The mixture was vortexed and incubated in the dark at room temperature for 30 min. Absorbance was measured at 517 nm using a microplate reader (SynergyMx, BioTek Instruments, USA), following the methodology described by Li et al. [23]. Analyses were performed in triplicate using independent extracts.

For the ABTS assay, the ABTS•^+^ radical cation was generated according to the method of Larrauri et al. [24] by reacting 5 mL of ABTS stock solution (7 mM) with 88 μL of potassium persulfate (140 mM). The resulting solution was left in the dark at room temperature for 16 h to ensure complete radical formation. It was then diluted with ethanol (98% *v*/*v*) to obtain an absorbance of 0.705 ± 0.02 at 735 nm, measured with a spectrophotometer (Shimadzu UV-1800, Shimadzu Corporation, Kyoto, Japan).

For sample analysis, 100 μL of the extract (LS or OMCS film) was added to 1.5 mL of the ABTS•^+^ working solution. After 6 min of reaction at room temperature, the absorbance was recorded at 735 nm, following the method of Li et al. [23]. Ethanol was used as the blank for absorbance adjustment. The radical scavenging activity for both assays was calculated according to Equation (9):

(9) DPPH/ABTS:Inhibition (%)=[(A_s−A_e)/A_s]×100

where A_s_ is the absorbance of the control solution (without extract), and A_e_ is the absorbance in the presence of the extract.

#### 2.4.11. Soil Burial Degradation Test

The soil burial degradation of the OMCS films was evaluated over 35 days under laboratory conditions using the soil burial method, following the procedure described by Reshmy et al. [25]. The soil was obtained from a local supplier (Fortaleza, CE, Brazil), homogenized, and placed in plastic containers. No prior physicochemical characterization of the soil (e.g., pH, moisture content, or organic matter) was performed.

Film specimens (2 × 1 cm) were weighed (P_1_) and buried at a depth of approximately 10 cm to ensure adequate exposure to soil microorganisms. The containers were maintained under ambient laboratory conditions, without active control of soil moisture or temperature during the experimental period. Visual changes were documented photographically. Samples were collected at 7, 14, 21, 28, and 35 days, carefully retrieved, gently washed to remove adhering soil particles, and dried in a forced-air oven (Marconi MA 035, Piracicaba, SP, Brazil) at 70 °C for 24 h. The dried samples were then reweighed (P_2_). Analyses were performed in triplicate (*n* = 3), and no positive or negative controls were included in the assay. The percentage of weight loss (%WL) was calculated according to Equation (10):

(10) $$Weight\;loss:\%WL=[(P_{1}-P_{2})/P_{1}]\times 100$$

where P_1_ is the initial dry weight of the sample, and P_2_ is the dry weight after burial.

#### 2.4.12. Statistical Analysis

All experiments were conducted in at least triplicate, and the results are expressed as mean ± standard deviation. The data were analyzed using analysis of variance (ANOVA) in Statistica (version 12.0; StatSoft Inc., Tulsa, OK, USA). When significant differences were detected, means were compared using Tukey’s post hoc test at the *p* < 0.05 significance level.

## 3. Results

### 3.1. Visual Appearance

The visual appearance of the OMCS films is shown in Figure 1A. All formulations produced continuous, flexible, and manageable films, without visible cracks or phase separation, with good compatibility among okra mucilage, corn starch, glycerol, and LS. Film F0 exhibited a lighter color, whereas incorporating LS led to progressive darkening of the films with increasing concentration. Changes in visual opacity were also observed, although not strictly linear, with LS-containing films generally appearing less transparent than F0. These visual changes are consistent with the instrumental results obtained for opacity and color parameters.

### 3.2. Rheological Behavior of FDDs

**Figure 2:**
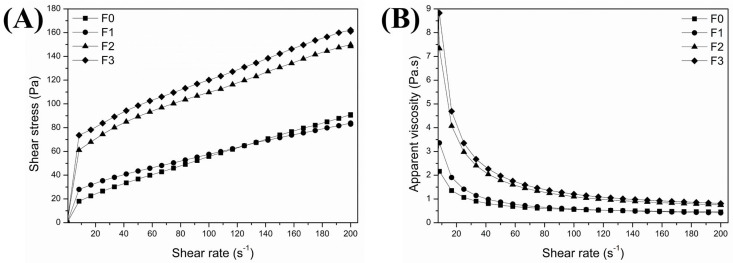
Effect of LS on the rheological behavior of FFDs: (**A**) shear stress as a function of shear rate and (**B**) apparent viscosity as a function of shear rate. F0 (0% LS), F1 (1% LS), F2 (2% LS), F3 (3% LS).

Table 1 and Figure 2 present the rheological parameters and flow behavior of the FFDs, which were fitted to the Power Law model and yielded excellent fits (R^2^ ≥ 0.98). All formulations exhibited pseudoplastic behavior (n < 1), consistent with systems containing high-molecular-weight polysaccharides such as okra mucilage and starch [8], as well as vegetable gums [10] and *Anchote* starch [26].

The consistency index (k) increased significantly (*p* < 0.05) with the addition of LS, rising from 4.34 ± 0.33 Pa·s^n^ in formulation F0 to 36.05 ± 1.81 Pa·s^n^ in formulation F3. This increase is likely due to enhanced intermolecular interactions between the polymeric matrix and the phenolic compounds present in LS. Similar trends have been reported for *Salvia macrosiphon* systems [10] and starch-based matrices [26], where bioactive compounds promoted increased matrix cohesion and densification.

Concomitantly, the flow behavior index (n) decreased from 0.56 ± 0.01 in formulation F0 to 0.24 ± 0.04 in formulation F2 and 0.25 ± 0.00 in formulation F3, indicating a pronounced increase in shear-thinning behavior. This reduction, together with the shear stress–shear rate profiles (Figure 2A), suggests the development of a more structured, shear-responsive network. Comparable behavior has been reported for systems containing plant mucilage and phenolic-rich extracts [8,25,27]. The presence of LS constituents, particularly phenolic and carbonyl compounds, may promote hydrogen bonding and hydrophobic interactions, thereby enhancing structural organization and stability [1,2].

In addition, the progressive reduction in pH observed after LS incorporation (Table 1) is consistent with the presence of organic acids in liquid smoke. It may also contribute to intermolecular interactions within the dispersions.

Overall, the results presented in Table 1 and Figure 2 demonstrate that LS significantly alters the rheological behavior of the FFDs, promoting increased apparent viscosity and greater internal structuring of the dispersions. These changes are expected to favor the formation of more homogeneous and cohesive films after drying, potentially influencing key properties such as thickness, water vapor permeability, and mechanical strength [8,10,26].

### 3.3. Physical and Mechanical Properties of OMCS Films

Table 2 summarizes the effect of liquid smoke (LS) on the thickness, moisture content, water solubility, water vapor permeability (WVP), and mechanical properties of OMCS films. Significant differences (*p* < 0.05) were observed among the formulations, indicating that LS influenced the structural organization and performance of the polymeric matrix.

#### 3.3.1. Thickness

The thickness values of the OMCS films are presented in Table 2. Despite the variation in LS concentration among the formulations, no significant differences were observed (*p* > 0.05). This behavior may be attributed to the relatively low variation in LS content, as well as the controlled casting conditions, which promote uniform film formation [8]. A slight, non-significant increase in average thickness with increasing LS concentration was observed, which is consistent with findings reported by Faisal et al. [1] for chitosan-based coatings incorporated with LS.

#### 3.3.2. Moisture Content

The moisture content of the OMCS films decreased gradually as the LS concentration increased. This reduction may be attributed to interactions between phenolic and carbonyl compounds present in LS and the hydroxyl groups of polysaccharides (okra mucilage and starch), which may reduce water retention through modifications in intermolecular interactions within the polymeric matrix [10].

Similar trends have been reported in LS-incorporated systems, including edible coatings applied to tofu [9] and chitosan-based films [1], where the incorporation of phenolic-rich extracts reduced moisture affinity.

#### 3.3.3. Water Solubility

A significant increase in water solubility (*p* < 0.05) was observed with increasing LS concentration, rising from 43.60 ± 3.83% in formulation F0 to 53.23 ± 0.78% in formulation F3. This behavior suggests that LS incorporation altered the structural organization of the polymeric matrix and affected its stability during water immersion.

Phenolic compounds are known to enhance intermolecular interactions in polysaccharide-based systems; however, their effects on film solubility are influenced by the organization of the polymer matrix, hydrogen bonding, and the overall composition of the polymer network. Araújo et al. [8] reported that the structural organization of okra mucilage–starch films significantly affects water-related properties. Meanwhile, Reshmy et al. [25] emphasized that additives rich in phenolic compounds can modify polymer interactions and enhance matrix stability. Additionally, Teseme et al. [26] demonstrated that incorporating functional compounds into starch-based films can alter water-interaction behavior, depending on the matrix’s composition and structural arrangement. According to Pan et al. [28], the incorporation of phenolic compounds can influence the hydrophilicity, microstructure, and intermolecular interactions of polysaccharide-based films, contingent on the matrix composition and the conditions used for film formation.

Since the films were dried prior to the solubility assay, the increase in solubility observed in this study is more likely attributable to changes in the structural stability of the dried matrix than to differences in moisture content. In addition, LS incorporation may have favored the release of water-soluble, low-molecular-weight compounds during immersion, contributing to the higher solubility values observed at higher LS concentrations.

#### 3.3.4. Water Vapor Permeability (WVP)

Table 2 presents the WVP of OMCS films incorporating LS. The values ranged from 1.05 ± 0.05 g·mm·m^−2^·h^−1^·kPa^−1^ in formulation F0 to 0.87 ± 0.02 g·mm·m^−2^·h^−1^·kPa^−1^ in formulation F3, showing significant differences among formulations (*p* < 0.05). A progressive decrease in WVP was observed as LS concentration increased, indicating improved barrier performance; however, formulation F1 deviated from this trend.

This deviation may be associated with the incomplete development of intermolecular interactions between LS components and the polymeric matrix at lower concentrations, resulting in a less compact and homogeneous structure. As LS content increases, the progressive establishment of these interactions likely contributes to matrix densification and reduced water vapor transmission. These values are relatively low compared to those typically reported for polysaccharide-based films, particularly starch-based systems, which generally exhibit equal or higher permeability depending on composition and processing conditions [29,30].

Water vapor permeability in polymeric films is governed by both the solubility of water in the matrix and its diffusion through the polymer network. A reduction in film hygroscopicity decreases the solubility coefficient, while increased matrix density and structural cohesion limit the diffusion coefficient [29]. Consequently, more compact and homogeneous matrices hinder water vapor transport, resulting in enhanced barrier properties [30].

The observed decrease in WVP may be attributed to changes in matrix organization and intermolecular interactions promoted by LS incorporation, driven by intermolecular interactions between the phenolic and carbonyl groups of LS and the hydroxyl groups of mucilage and starch polysaccharides. These interactions likely reduce polymer chain mobility and restrict water diffusion through the film. Similar trends have been reported for LS-containing systems, including *Salvia macrosiphon*-based films [10] and chitosan films [1]. Both showed significant reductions in WVP as LS content increased.

Additionally, Araújo et al. [8] reported low WVP values for okra mucilage–starch films, attributed to the formation of cohesive and homogeneous polymer networks. More recent studies by Teseme et al. [26] have demonstrated that incorporating hydrophobic compounds into starch-based films reduces water diffusion and enhances structural stability. Likewise, Reshmy et al. [25] highlighted that phenolic-rich plant extracts can act as functional modifiers, improving resistance to water vapor transmission.

Overall, the reduction in WVP observed in this study suggests that LS simultaneously affects both the solubility and diffusion components of water transport, leading to films with improved water vapor barrier performance and modified structural organization. These findings support the potential of LS as a natural functional additive to enhance the barrier properties of OMCS films, particularly for active packaging of moisture-sensitive foods.

#### 3.3.5. Mechanical Properties

The mechanical performance of the OMCS films is summarized in Table 2. A progressive increase in tensile strength (TS) was observed with LS addition, ranging from 26.30 ± 0.50 MPa in formulation F0 to 40.54 ± 1.46 MPa in formulation F3, with statistically significant differences among samples (*p* < 0.05). This strengthening effect is consistent with reports that phenolic and carbonyl constituents of LS can interact with hydroxyl-rich polysaccharide matrices, promoting additional intermolecular interactions and contributing to a more cohesive network [1,10]. In polysaccharide-based films, enhanced chain packing and reduced microdefects have been associated with increased mechanical resistance, which may also explain the trend observed here, as reported by Dąbrowska et al. [27].

In contrast, elongation at break (E%) showed a slight but significant reduction, decreasing from 5.22 ± 1.20% in formulation F0 to 4.12 ± 0.24% in formulation F3. The decline in deformability suggests restricted molecular mobility, likely due to the formation of a denser polymer network upon incorporation of LS constituents [26]. Similar behavior has been reported for films containing phenolic compounds, where structural reinforcement generally increases stiffness while maintaining matrix integrity [25].

The addition of LS resulted in films with increased tensile strength and a moderate decrease in elongation, indicating the formation of a stiffer and more resistant matrix. This balance between rigidity and flexibility is advantageous for packaging applications that require mechanical durability during handling, transportation, and storage.

#### 3.3.6. Contact Angle (CA)

**Figure 3:**
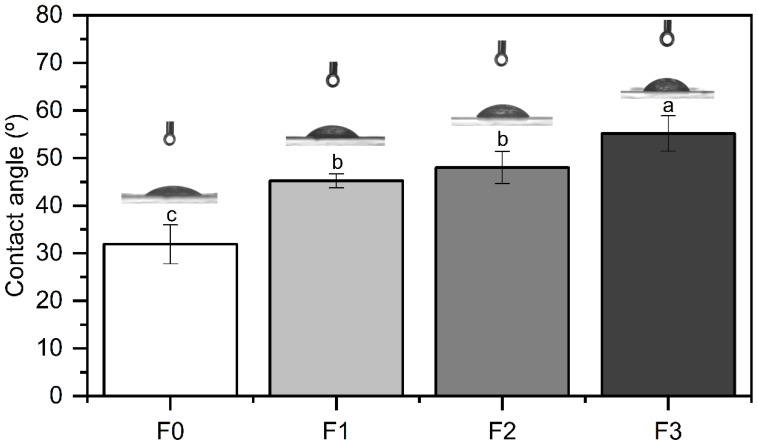
Contact angle values and visual appearance of water droplets on OMCS films containing increasing concentrations of LS: F0 (0% LS), F1 (1% LS), F2 (2% LS), and F3 (3% LS). Bars sharing the same letter are not significantly different according to Tukey’s multiple-comparison test (*p* < 0.05).

The surface wettability of the OMCS films containing LS is shown in Table 2 and Figure 3. An increase in LS concentration led to visibly reduced spreading of the water droplet, accompanied by higher CA values. Quantitatively, the CA increased from 31.89 ± 4.09° in formulation F0 to 55.18 ± 3.72° in formulation F3, with statistically significant differences among samples (*p* < 0.05). This trend indicates a progressive decrease in surface hydrophilicity after LS incorporation into the polymer matrix.

The increase in CA may be associated with modifications in surface energy induced by the phenolic and carbonyl constituents of LS, which can reduce surface polarity and promote the formation of more hydrophobic domains at the film–air interface. Similar enhancements in hydrophobicity have been reported for LS-containing chitosan films and coatings, where aromatic compounds from pyrolysis were shown to improve resistance to water spreading [1,10]. In polysaccharide-based systems, plant-derived phenolic compounds have been shown to decrease wettability by promoting tighter molecular packing and reducing the density of hydrophilic groups on the surface [25,26].

The behavior observed here is also consistent with previous findings on okra mucilage–starch films, which exhibit good cohesion and a uniform microstructure, characteristics that facilitate the formation of continuous and smooth surfaces with reduced water affinity [8].

Thus, LS incorporation increased surface hydrophobicity and improved moisture resistance, although the overall interaction of the films with water also depended on matrix organization and structural stability during immersion. This enhancement is advantageous for biodegradable packaging applications, as reduced water absorption improves stability and protects moisture-sensitive foods.

### 3.4. Optical Analyses

The color parameters (L*, a*, b*), whiteness index (WI), yellowness index (YI), opacity, and transparency of OMCS films are summarized in Table 3. Overall, increasing LS concentration influenced the L* values and promoted darker coloration in the LS-containing films, although formulation F1 exhibited behavior distinct from the overall trend. Concurrently, both a* and b* values increased, reflecting a shift toward a more yellowish and slightly reddish appearance in the LS-containing films.

This chromatic change is consistent with the optical behavior of phenolic and carbonyl compounds generated during biomass pyrolysis, which absorb visible light and impart brownish coloration to polysaccharide-based materials [10]. Similar trends have been reported for starch films and chitosan-based coatings incorporating LS, where the rise in a* and b* values was attributed to the presence of aromatic constituents and volatile pigments [1,2]. Gani et al. [9] likewise observed yellowish coloration on LS-treated tofu surfaces, suggesting the deposition of phenolic compounds.

Changes in WI and YI values further indicate that LS incorporation affected the color intensity and visual appearance of the films, although some parameters did not follow a strictly linear trend among formulations. Although a darker appearance may limit applications requiring high transparency or neutral color, this phenomenon can also reflect the presence of bioactive phenolic compounds, which contribute to enhanced antioxidant performance relevant to active food packaging [1,26].

Opacity values increased progressively with LS incorporation, while transparency decreased across all formulations (Table 3, Figure 1). This behavior likely results from both the intrinsic color of LS and the presence of phenolic compounds incorporated into the polymeric matrix. Similar increases in opacity were previously reported in ginger starch films containing liquid coconut shell smoke [2]. Enhanced opacity may also reflect higher optical density and possible modifications in surface microstructure or roughness arising from LS–polymer interactions. According to Amininasab et al. [10] and Faisal et al. [1], increased opacity can be advantageous in packaging designed for light-sensitive foods, as it reduces light transmission and mitigates photo-oxidative degradation.

The incorporation of LS resulted in significant changes in the film’s optical properties, leading to darker coloration and reduced transparency. These effects align with the chemical properties of LS and the structural modifications within the polysaccharide matrix. Such alterations may benefit the development of active OMCS-based packaging materials by enhancing antioxidant potential and improving light-barrier capacity.

### 3.5. Fourier-Transform Infrared Spectroscopy (FTIR)

**Figure 4:**
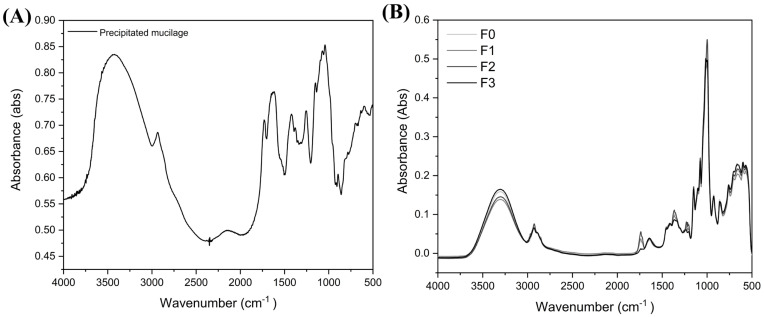
(**A**) FTIR spectrum of isolated okra mucilage; (**B**) FTIR spectra of OMCS films containing increasing concentrations of LS. F0, F1, F2, and F3 correspond to films containing 0%, 1%, 2%, and 3% LS, respectively.

The FTIR spectra of precipitated okra mucilage and the corresponding OMCS films containing LS are presented in Figure 4. In the spectrum of the isolated mucilage (Figure 4A), the broad band centered around 3441 cm^−1^ corresponds to O–H stretching vibrations typical of hydroxyl-rich polysaccharides, confirming the hydrophilic nature of the material. The peak at 2934 cm^−1^ is attributed to C–H stretching of aliphatic groups present in the sugar residues. A weak signal near 1637 cm^−1^ is commonly associated with bound water and the asymmetric stretching of carboxylate groups, consistent with the presence of galacturonic acid in okra mucilage. The region between 1000 and 1200 cm^−1^, dominated by C–O and C–O–C stretching from pyranose ring vibrations and glycosidic bonds, showed a strong band at 1075 cm^−1^, characteristic of galactose-, rhamnose-, and galacturonic-acid-based structures [31,32]. Overall, these spectral features confirm the carbohydrate composition of the mucilage.

In the spectra of the films (Figure 4B), the broad O–H band remained evident, although slight variations in shape and intensity may reflect hydrogen-bond rearrangements involving starch, mucilage, glycerol, and absorbed water [33]. A noticeable band in the 1600 cm^−1^ region increased with LS incorporation, consistent with C=O stretching of carbonyl and carboxylate groups present in acetic acid and related volatile components of LS [34]. Additionally, bands detected between 700 and 900 cm^−1^ correspond to out-of-plane bending of aromatic C–H bonds, which are typical of phenolic compounds found in pyrolysis-derived smoke condensates [35].

Overlaying the spectra of formulations F0–F3 revealed progressive increases in absorbance in the regions assigned to LS-related functional groups. Sample F3 exhibited the highest intensities near ~1600 cm^−1^ and ~780 cm^−1^, indicating a greater presence of carbonyl- and aromatic-containing constituents. The monotonic increase in band intensity across the formulations demonstrates concentration-dependent incorporation of LS into the film matrix, as reflected in the spectral signatures of phenols, cresols, and related compounds.

### 3.6. Surface Morphology

Figure 1B shows the surface morphology of the OMCS films containing increasing concentrations of LS. Film F0 exhibited a relatively rough surface with visible pores and fissures, suggesting limited compatibility between starch and mucilage. Similar structural discontinuities have been reported in polysaccharide films lacking additional interacting compounds [2].

At low LS concentration (F1), the surface appeared more compact, with a noticeable reduction in pores. This improvement may reflect enhanced intermolecular interactions among LS constituents, particularly phenolic compounds, and the polysaccharide matrix, contributing to increased structural cohesion. Such densification effects have also been described in protein- and polysaccharide-based films incorporating smoke condensates [36].

The intermediate LS concentration (F2) produced a more homogeneous, continuous surface, consistent with a balanced interaction among mucilage, starch, and the functional groups in LS. Comparable morphological stabilization has been observed in systems containing optimized proportions of smoke-derived phenolic additives [35].

In contrast, the highest LS concentration (F3) led to the formation of granular structures and surface crystallites. These features may indicate partial matrix saturation and localized heterogeneities consistent with phase separation at high LS loading, which can disrupt film uniformity. Similar phenomena have been reported at elevated levels of phenolic additives, where excessive incorporation compromises structural regularity [35].

Thus, the SEM analysis indicates that LS has a concentration-dependent effect on the film morphology. Moderate LS levels promote surface compaction and structural uniformity, whereas excessive concentrations lead to surface heterogeneity and potential disruption of the polymer network.

The AFM images (Figure 1C) provided complementary information regarding the surface topography of the OMCS films. Consistent with the SEM observations, the control film (F0) exhibited greater height variations and a more pronounced three-dimensional topography. With increasing LS concentration, the surface profiles became progressively smoother, particularly in F1 and F2, indicating a more homogeneous surface organization.

The three-dimensional AFM reconstructions further supported this behavior, showing less pronounced peaks and valleys in the LS-containing films compared with the control formulation. Similar applications of AFM have been reported for starch- and mucilage-based films, where the technique was used to evaluate surface homogeneity and topographical changes associated with film composition [37,38].

Although localized irregularities were observed in F3, the AFM images suggested that these features remained restricted to specific regions of the surface. Overall, the AFM results corroborated the SEM observations and indicated that LS promoted concentration-dependent modifications in the surface organization of OMCS films.

### 3.7. Antioxidant Activity and TEP

**Figure 5:**
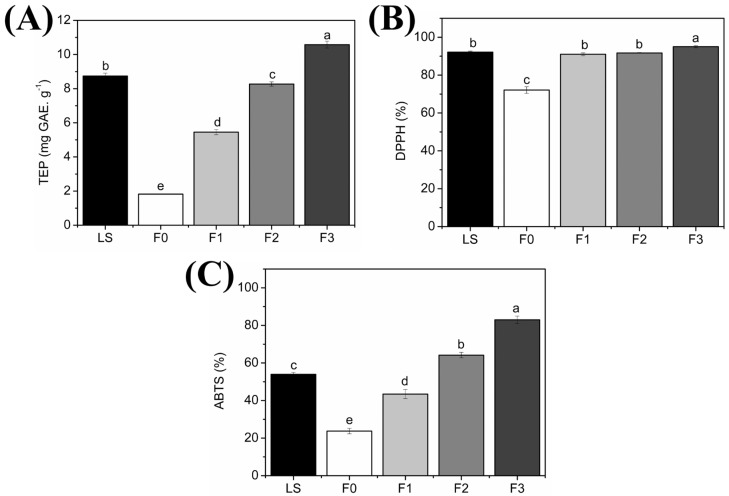
(**A**) Total extractable polyphenol content (TEP), (**B**) DPPH radical scavenging activity, and (**C**) ABTS radical scavenging activity of LS and OMCS films. F0, F1, F2, and F3 correspond to films containing 0%, 1%, 2%, and 3% LS, respectively. Error bars represent standard deviation. Different lowercase letters indicate significant differences among formulations according to Tukey’s test (*p* < 0.05).

The antioxidant capacity of OMCS films was assessed using the ABTS and DPPH assays, while TEP values served as an indicator of the incorporation of phenolic constituents into the polymer matrix (Figure 5). For comparison purposes, the LS used as an additive was also analyzed independently and exhibited high TEP (~8.8 mg GAE g^−1^) and strong antioxidant activity (~92% DPPH; ~55% ABTS). These results confirm its high concentration of phenolic and carbonyl compounds formed during biomass pyrolysis, as previously reported [1,2].

In contrast, the OMCS film F0 showed markedly lower TEP (~2.0 mg GAE g^−1^) and limited antioxidant activity (~72% DPPH; ~25% ABTS), indicating that a matrix composed solely of okra mucilage and starch contributes modestly to antioxidant capacity, similar to observations in starch films without LS [2].

Incorporation of LS resulted in a significant (*p* < 0.05) and concentration-dependent increase in TEP, rising from ~2.0 mg GAE g^−1^ in formulation F0 to ~10.5 mg GAE g^−1^ in formulation F3. A proportional rise in ABTS inhibition was also observed, increasing from ~25% in formulation F0 to ~82% in formulation F3. This trend demonstrates that ABTS is particularly sensitive to the total phenolic content incorporated into the matrix, as previously noted in polysaccharide films containing smoke condensates [1].

The DPPH results, however, showed a more moderate increase. Antioxidant activity rose sharply from ~72% in formulation F0 to ~95% in formulation F1 but plateaued at higher LS concentrations remaining between ~92 and 94% in formulations F2 and F3. This stabilization suggests that some phenolic compounds may become partially immobilized or less accessible within the polysaccharide network due to interactions with the hydroxyl groups of starch and mucilage. Similar behavior has been described by Faisal et al. [1], Reshmy et al. [25] and Teseme et al. [26], who reported that while ABTS reflects the total pool of extractable phenolics, DPPH is more dependent on the immediate accessibility of reactive compounds.

In summary, the combined analysis of TEP, ABTS, and DPPH confirms that LS is the primary contributor to the films’ antioxidant activity. The polymer matrix significantly influences the release and availability of these compounds, highlighting LS as an effective functional additive for biodegradable films used in antioxidant-active food packaging applications. This aligns with the growing global trend in research toward incorporating phenolic-rich plant-derived functional additives into bio-based packaging systems [11].

### 3.8. Soil Burial Degradation

**Figure 6:**
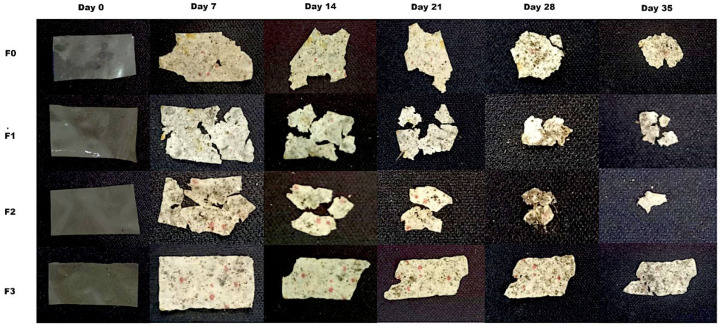
Photographic images of OMCS films subjected to a soil burial test for 35 days. Images were recorded at Day 0 and after 7, 14, 21, 28, and 35 days of soil burial. F0, F1, F2, and F3 correspond to films containing 0%, 1%, 2%, and 3% LS, respectively. The color differences observed among the samples correspond to the original film formulations and the visual changes occurring during the soil burial degradation process.

Degradability is a key requirement for bio-based films intended as sustainable alternatives to conventional plastic packaging. The soil burial test was conducted over 35 days (Figure 6), during which progressive degradation of the OMCS films was observed. All samples exhibited visible signs of structural deterioration, including surface cracking, shrinkage, embrittlement, and fragmentation. These visual changes are commonly associated with biodeterioration, which precedes more advanced degradation stages [39]. Notably, formulations F1 and F2 exhibited earlier degradation, with clear morphological disruption observed as early as Day 7 and Day 14, respectively. These observations suggest that incorporating LS into the polysaccharide matrix does not hinder degradation and may even influence degradation kinetics under soil burial conditions.

**Figure 7:**
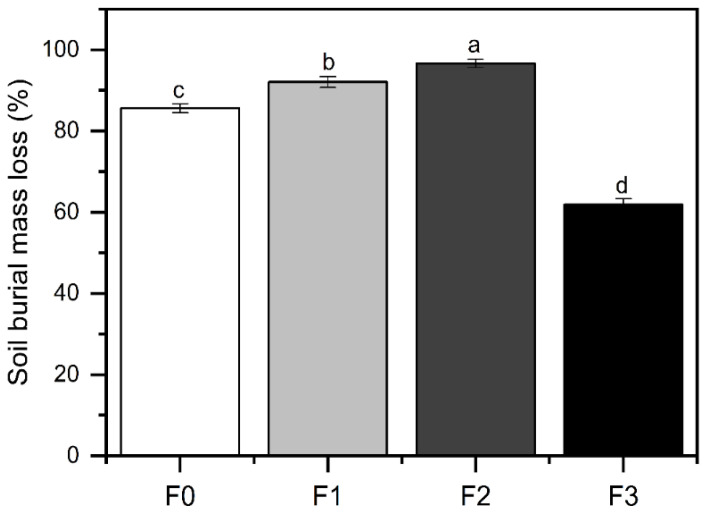
Final mass loss of OMCS films after 35 days of soil burial. F0, F1, F2, and F3 correspond to films containing 0%, 1%, 2%, and 3% LS, respectively. Error bars represent standard deviation. Different lowercase letters indicate significant differences among formulations according to Tukey’s test (*p* < 0.05).

The mass loss values obtained under soil conditions (Figure 7) ranged from 61.84% to 96.64%, consistent with the expected behavior of starch-containing films, as their high water affinity facilitates enzymatic and microbial degradation in soil environments [26]. Similar behavior has been reported for cassava starch-based composites buried under indoor soil conditions, in which water sorption promoted microbial growth and accelerated weight loss of the materials [40]. Although all formulations showed degradation under soil burial conditions, degradation increased with increasing LS incorporation up to F2; however, this pattern was not maintained at the highest LS concentration (F3).

It should be noted that gravimetric mass loss in soil burial assays may include not only microbial degradation of the polymeric matrix but also the leaching of soluble components such as glycerol and low-molecular-weight phenolic compounds, and therefore does not exclusively represent complete mineralization of the material [39]. Thus, the present results are more appropriately interpreted as an indicator of degradation behavior under soil burial conditions.

This reduction in degradation rate for F3 is consistent with the structural, physicochemical, and bioactive modifications previously demonstrated in this study. Formulation F3 exhibited the lowest WVP (0.8795 g·mm·m^−2^·h^−1^·kPa^−1^), the highest CA (55.18°), and the greatest TS (40.54 MPa) (Table 2), indicating the formation of a more cohesive and hydrophobic matrix. These characteristics limit moisture penetration into the polymer network, a factor essential for microbial colonization and enzymatic activity in soil systems. Lucas et al. [39] emphasized that polymer degradation in natural environments commonly depends on preliminary abiotic effects, especially water diffusion into the matrix and structural weakening prior to microbial assimilation.

Furthermore, the antioxidant analyses (TEP, ABTS, and DPPH; Figure 5) demonstrated substantial incorporation of LS-derived phenolic compounds, which are known for their antioxidant properties [2,27]. The presence of these bioactive constituents may further reduce microbial activity on the film surface and decelerate the initial stages of degradation.

Therefore, the lower degradation observed for F3 does not indicate a loss of degradability but rather reflects greater structural stability resulting from matrix reinforcement, enhanced hydrophobicity, and the incorporation of bioactive LS components. Overall, the OMCS films remained susceptible to degradation under soil burial conditions, although the degradation kinetics were influenced by LS concentration. It is acknowledged that the absence of positive and negative controls limits the ability to confirm soil biological activity and the assay’s discriminatory power. Future studies should include standardized controls and controlled environmental conditions to better align with methods such as ASTM D5988-18 [41] and ISO 17556:2019 [42].

## 4. Conclusions

The incorporation of LS into OMCS films led to concurrent improvements in their structural, physicochemical, and functional properties. The results of the study indicate that phenolic constituents of LS interact with hydroxyl groups within the polysaccharide matrix, contributing to the formation of a more cohesive network. These structural changes resulted in higher mechanical strength, increased contact angle and opacity, and improved water vapor barrier performance, along with a moderate increase in water solubility, indicating that the effect of LS on matrix organization depends on the balance between intermolecular interactions and structural arrangement. The TEP, ABTS, and DPPH assays confirmed that LS is the primary source of antioxidant activity in the films, whereas the polymeric matrix modulates the accessibility of these compounds.

Under soil burial conditions, all formulations showed substantial mass loss, indicating susceptibility to degradation. However, higher LS concentrations were associated with slower degradation behavior, likely due to increased hydrophobicity and enhanced structural stability. In summary, the role of LS as a structural and functional additive entailed two primary functions: enhancing matrix integrity and imparting antioxidant properties. This multifaceted contribution of LS expands the potential applications of OMCS films for active food packaging. Future studies should further investigate the antimicrobial potential of these films to broaden their applicability in food preservation systems.

Further research is needed to assess the ecotoxicological effects of degradation products released during soil biodegradation to enhance our understanding of the environmental safety of LS-containing films.

## Figures and Tables

**Table 1:** Effect of LS on the rheological parameters and pH of the FFDs.

LS	Rheological Parameters			pH
	k (Pa·s^n^)	n	R^2^	
F0	4.34 ± 0.33 ^b^	0.56 ± 0.01 ^a^	0.98	6.14
F1	9.33 ± 0.87 ^b^	0.40 ± 0.01 ^b^	0.99	4.64
F2	35.41 ± 6.92 ^a^	0.24 ± 0.04 ^c^	0.99	4.07
F3	36.05 ± 1.81 ^a^	0.25 ± 0.00 ^c^	0.99	3.94

Means sharing the same letter within a column do not differ significantly according to Tukey’s multiple-comparison test (*p* < 0.05). F0, F1, F2, and F3 correspond to formulations containing 0%, 1%, 2%, and 3% LS, respectively.

**Table 2:** Effect of LS on the physical, barrier, and mechanical properties of OMCS films.

Analysis	LS				*p*-Value
	F0	F1	F2	F3	
Thickness (µm)	33.2250 ± 2.4001 ^a^	34.5000 ± 1.4086 ^a^	34.5250 ± 1.0510 ^a^	34.8250 ± 0.9039 ^a^	0.051
Moisture (%)	6.9891 ±1.0639 ^a^	6.2727 ±0.9791 ^a^	6.7000 ±1.0639 ^a^	6.4914 ±0.4028 ^a^	0.002
Solubility (%)	43.6046 ± 3.8320 ^b^	46.3021 ±3.4883 ^ab^	47.3585 ±2.4509 ^ab^	53.2319 ±0.7895 ^a^	0.016
WVP (g·mm·m^−2^·h^−1^·kPa^−1^)	1.0598 ± 0.0500 ^a^	1.5568 ± 0.3995 ^a^	0.9332 ±0.0674 ^a^	0.8795 ±0.0293 ^b^	0.0003
Tensile Strength (MPa)	26.3057 ± 1.8760 ^b^	28.0925 ± 2.1936 ^b^	29.6700 ± 3.9222 ^b^	40.5460 ± 1.6405 ^a^	0.0000
Elongation (%)	5.2285 ± 1.2084 ^ab^	5.8475 ± 0.7832 ^a^	3.6457 ±0.7302 ^c^	4.1280 ±0.2457 ^bc^	0.0002
Contact angle (°)	31.8900 ± 4.0964 ^c^	45.2300 ± 1.4689 ^b^	48.0100 ± 3.3692 ^b^	55.1800 ± 3.7210 ^a^	0.0000

Means sharing the same letter within a column do not differ significantly according to Tukey’s multiple-comparison test (*p* < 0.05). F0, F1, F2, and F3 correspond to films containing 0%, 1%, 2%, and 3% LS, respectively.

**Table 3:** Effect of LS on the optical properties of OMCS films.

Properties	LS				*p*-Value
	F0	F1	F2	F3	
L	94.5000 ± 0.2851 ^b^	95.4100 ± 0.2749 ^a^	94.6066 ± 0.111 ^b^	94.3500 ± 0.3124 ^b^	0.0042
a*	−1.0600 ± 0.0173 ^b^	−1.1866 ± 0.0208 ^a^	−1.0633 ± 0.0152 ^b^	−1.0866 ± 0.0251 ^b^	0.0001
b*	3.8800 ± 0.0435 ^d^	5.9633 ± 0.1026 ^a^	4.6066 ± 0.0378 ^c^	5.1133 ± 0.1913 ^b^	0.0000
ΔE	0.0000	2.2895 ± 0.0218 ^a^	0.7401 ± 0.0393 ^c^	1.2665 ± 0.2146 ^b^	0.0000
Whiteness Index	78.3205 ±0.4518 ^a^	58.4331 ±1.5175 ^d^	72.2535 ± 0.3762 ^b^	66.9981 ± 2.1134 ^c^	0.0000
Yellowness Index	22.7605 ± 0.5206 ^d^	53.2612 ± 1.9785 ^a^	32.0465 ± 0.5210 ^c^	39.6285 ± 2.9797 ^b^	0.0000
Opacity	4.7355 ± 0.6922 ^b^	6.8100 ± 0.5256 ^ab^	7.3473 ± 1.4713 ^a^	8.8346 ± 0.2391 ^a^	0.0030
Transparency	2111.864 ± 108.3661 ^a^	1857.591 ± 17.4590 ^b^	1466.380 ± 27.1404 ^c^	1081.004 ± 57.9582 ^d^	0.0000

Means sharing the same letter within a column do not differ significantly according to Tukey’s multiple-comparison test (*p* < 0.05). F0, F1, F2, and F3 correspond to films containing 0%, 1%, 2%, and 3% LS, respectively.

## Data Availability

The raw data supporting the conclusions of this article will be made available by the authors on request.
